# P03-007 - Mevalonate kinase gene in Behçet’s disease

**DOI:** 10.1186/1546-0096-11-S1-A202

**Published:** 2013-11-08

**Authors:** D Arslan Tas, E Erken, F Yıldız, S Dinkçi, H Sakallı

**Affiliations:** 1 Rheumatology-Immunology Department, ADANA, Turkey; 2 Cukurova University, Faculty Of Medicine, ADANA, Turkey; 3 Internal Medicicne, Medline Hospital, ADANA, Turkey

## Introduction

Genetics is suggested to play role in the development of Behçet’s disease (BD). Shared phenotipic features requires an approach to differential diagnosis from periodic febrile syndromes.

## Objectives

We planned to study for mevalonate kinase (MVK) as a candidate for a susceptibility gene for Behçet’s disease.

## Methods

Consecutive Behçet patients and apperently healthy subjects were included. Severity score of Behçet disease was calculated. Genotyping of mevalonate kinase gene was done by polymerase chain reaction /sequence-based typing technique.

## Results

50 BD patients (median age: 38.30±11.06 years) and 51 controls (median age: 33.88±12.47 years) were recruited. Three types of mutations have found. First: A single nucleotide polymorphism (SNP) c.769-38C>T(rs35191208) in 21 of 50 BD patients and in 15 of 51 controls. Both groups were comparable for the frequency of c.769-38C>T (p>0,05). In all of the cases with c.769-38C>T, a second SNP: c885+24G>A(rs2270374) was also present (previously reported to be in linkage disequilibrium with the first SNP). Third SNP: c.769-7T>G(rs104895331) was found in 3 of 50 BD patients and in 1 of the control group. We found this SNP together with c769-38C>T and c.885+24G>A. The neurological involvement was found to be more frequent in the BD patients with c.769-38 C>T when compared to the BD patients without this polymorphism (p:0,012).

## Conclusion

Our results suggested that the effects of MVK mutations in Behçet’s disease could be an additional genetic susceptibility factor for the patients with neurological involvement. However these results need confirmation in larger study populations and in different ethnic groups.

## Competing interests

None Declared.

